# The association of early feeding practices with eating behaviors and maternal indulgent feeding behaviors among Saudi preschoolers

**DOI:** 10.3389/fpsyg.2023.1126687

**Published:** 2023-08-01

**Authors:** Rana H. Mosli, Hebah A. Kutbi

**Affiliations:** Clinical Nutrition Department, Faculty of Applied Medical Sciences, King Abdulaziz University, Jeddah, Saudi Arabia

**Keywords:** eating behaviors, feeding, preschool, fruit juice, baby bottle

## Abstract

Evidence from previous work suggest that feeding practices during the first years of life may influence the development of eating behaviors later in childhood. Early feeding practices may also predict later maternal feeding behaviors. Mothers from an Arab/Middle Eastern background may adopt unique early feeding practices. The objective of this study was to examine the association of offering fruit juice and date syrup-milk mixture in a baby bottle during infancy with: 1) Child eating behaviors during preschool years; 2) Maternal indulgent feeding practices during preschool years. Mothers of preschoolers (*n* = 115) completed questions adopted from the Children’s Eating Behavior Questionnaire (CEBQ), Child Feeding Questionnaire-Arabic (CFQ-A), as well as questions assessing early feeding practices and demographic characteristics. Unadjusted and adjusted logistic regression models were tested to examine the association of early feeding practices with child eating behaviors and maternal indulgent feeding behaviors. Odds ratios (ORs) and respective 95% confidence intervals (CI) were derived. Adjusting for covariates, children who were ever offered fruit juice in a baby bottle as infants were more likely to have high food responsiveness at preschool, compared to children who were not (OR = 2.93, 95% CI = 1.16, 7.42). Mothers who reported ever offering fruit juice in a baby bottle when their children were infants were more likely to adopt greater use of food as a reward when their children were at preschool (OR = 3.25, 95% CI = 1.22, 8.68). Early feeding practices are associated with child eating and maternal feeding behaviors later in childhood. Future longitudinal studies are needed to further establish these associations. Findings from this study can help inform community awareness and education programs to prevent maladaptive feeding practices and promote appropriate feeding strategies throughout childhood.

## 1. Introduction

The first few years of life are critical in determining behaviors around food and lifelong risk of excess adiposity (Birch and Fisher, 1998; Birch and Davison, 2001; Scaglioni et al., 2018; Dubois et al., 2022). Children develop their eating behaviors through an array of innate and acquired traits; both genetic and environmental factors (including the home environment) have been shown to affect the development of eating behaviors in children (Scaglioni et al., 2018; Kutbi et al., 2019). Family functioning and interactions among family members around food, as well as parenting practices, may be among the main factors in the home environment influencing child eating behaviors (Birch and Fisher, 1998; Birch and Davison, 2001; Scaglioni et al., 2018; Kutbi et al., 2019; Mosli and Kutbi, 2022). For example, maternal feeding practices, such as using rewards for behavior and structured meal timing, have been found to be associated with food responsiveness and satiety responsiveness among preschool children (Jansen et al., 2018). Higher maternal restriction and monitoring of child’s eating have been linked to higher levels of emotional eating among children (Kröller et al., 2013), while responsive food parenting styles that are authoritative and sensitive may lead to more positive child eating behaviors, dietary patterns, and growth outcomes (Balantekin et al., 2020).

Evidence from previous work suggest that feeding practices during the first 2 years of life may play a role in shaping the development of eating behaviors and habits among children (Daniels et al., 2014; Nicklaus, 2017; Scaglioni et al., 2018; Switkowski et al., 2020). For example, among preschoolers, being fed directly from the mother’s breast during infancy was linked to higher satiety responsiveness (DiSantis et al., 2011), while being breastfed for less than 6 months was found to be associated with higher levels of food-approach behaviors (Ergang et al., 2021). Shorter breastfeeding duration was also linked to poorer satiety responsiveness in adolescence (Reyes et al., 2014). Although ample research has examined the relationship between breastfeeding and eating behaviors, the association of other early feeding practices with child eating behaviors during preschool years has been underdeveloped, particularly among non-Western cultures (Scaglioni et al., 2018). Mothers from an Arab/Middle Eastern background may adopt unique early feeding practices (Alzaheb, 2017; Campoy et al., 2018); recent data concerning early feeding behaviors have shown a high proportion of Saudi mothers (about half) offering fruit juice (FJ) and date syrup-milk mixture (DSMM) in a baby bottle (Mosli, 2022). FJ should be limited during early years and should not be offered in baby bottles or toddler cups due to risk of overconsumption and related negative consequences (Imamura et al., 2015; Heyman and Abrams, 2017), which include replacing important food sources and reduction of intake of certain nutrients, short stature, exceeding sugar requirement, diarrhea, tooth decay, pneumonia, and overweight (Watkins et al., 1979; Hyams and Leichtner, 1985; Dennison et al., 1997; Behrendt et al., 2001; Shefferly et al., 2016; Murray, 2020) Although the association of offering FJ and DSMM in a baby bottle with child eating behaviors during preschool years is unknown, findings from a previous study involving a United States sample suggest that delaying juice introduction is associated with better diet quality at age three (Switkowski et al., 2020). Since maladaptive complimentary feeding patterns may negatively influence the development of appetite control and learnt food pleasure among children (Nicklaus, 2017), and the period of complementary food introduction may be important in shaping children’s ability to self-regulate food intake (Balantekin et al., 2020), early introduction of sugary or sweet drinks may predict less favorable eating patterns among children. In the present study, we hypothesized that offering FJ and DSMM in a baby bottle during infancy is associated with higher level of food-approach and lower level of food-avoidant eating behaviors during preschool years.

Feeding practices that mothers employ when their children are infants may also be associated with maternal feeding behaviors later in life (Li et al., 2014); mothers encouraging their infants to empty their bottle during infancy were found to be more likely to encourage their children to empty their plates at age six (Li et al., 2014). Furthermore, breastfeeding duration has been found to be positively associated with maternal responsive feeding behaviors later in life (Ventura, 2017). However, the relationship between early feeding practices and feeding behaviors later in life has not been efficiently explored in Arab/Middle Eastern samples. Since mothers with Arab/Middle Eastern backgrounds may adopt unique feeding practices, such as offering FJ and DSMM in a baby bottle (Mosli, 2022), establishing associations with feeding behaviors later in life may be an important step in understanding population targets for future intervention programs and community awareness campaigns. In the present study, we hypothesized that offering FJ and DSMM in a baby bottle during infancy is associated with more indulgent feeding practices during preschool years.

The objectives of this study were to examine the association of offering FJ and DSMM in a baby bottle during infancy with: 1) Child eating behaviors during preschool years and 2) Maternal indulgent feeding practices during preschool years.

## 2. Methods

### 2.1. Participants

The study sample included mothers of preschoolers (*n* = 115) residing in the city of Jeddah, Saudi Arabia (SA). Eight preschools were chosen as sites of recruitment based on location and whether they were public (government-subsidized) or private schools. Two schools were included from the Northern area, two from the Southern area, two from the Eastern area, and two from the Western area. Of the eight preschools, four were public and four were private preschools. Following approval from the Education Administration in Jeddah, the research team sent a formal letter to the head of each preschool with a detailed description of the study protocol. Subsequently, preschoolers’ mothers were contacted through a sealed envelope placed in each child’s backpack. The envelope included a letter containing a brief description of the study and the consent form. Each class teacher was responsible for placing the envelopes in the backpacks. Members of the research team then contacted mothers who returned completed consent forms and were eligible to participate via telephone to complete the study questionnaire; questions and response options were read, and mothers’ answers were recorded.

Inclusion criteria for the study were that the mother is: 1) Saudi or a permanent resident of SA, 2) fluently speaks Arabic 3) has a healthy child, between the ages of three and 5 years old, with no history of serious medical conditions or food allergies, 4) resides with the child in the same household. Mothers who did not reside primarily with their children were excluded from the study.

Questions in the study questionnaire assessed child eating behaviors, indulgent feeding behaviors at preschool, early feeding practices and demographic characteristics. The study protocol was approved by the Unit of Biomedical Ethics at King Abdulaziz University (HA-02-J-008).

### 2.2. Measures

#### 2.2.1. Outcomes: child eating behaviors and indulgent feeding behaviors

Mothers completed questions that assessed child eating behaviors at the time of the study. These questions were adopted from the Children’s Eating Behavior Questionnaire (CEBQ). The CEBQ was developed and validated in the United Kingdom in a study involving preschoolers and school-aged children (Wardle et al., 2001). Subsequently, a study conducted among a group of Saudi preschoolers has shown evidence of validity and reliability of an Arabic version of the CEBQ (Alhamad, 2013). The CEBQ consists of 35 items that generate eight factors which are as follows: 1) emotional over eating (EOE), 2) emotional under eating (EUE), 3) food responsiveness (FR), 4) enjoyment of food (EF), 5) desire to drink (DD), 6) food fussiness (FF), 7) satiety responsiveness (SR), and 8) slowness in eating (SE). Response options of all items consist of a five-point Likert scale ranging from 1 = “never” to 5 = “always.” Factors are calculated as the mean of contributing items.

As previously employed in a study involving Saudi preschoolers (Mosli and Kutbi, 2022) and other non-Western samples (Alhamad, 2013; Mallan et al., 2013; Purwaningrum et al., 2020), modification of some of the CEBQ factors by excluding certain items was completed in order to improve internal consistency (Santos, 1999); one item was removed from each of the three factors “FR,” “EF,” and “SR.” From the FR scale, the item “even if my child is full up s/he finds room to eat his/her favorite food” was excluded. From the EF scale, the item “My child looks forward to mealtimes” was excluded. From the SR scale, the item “my child has a big appetite” was excluded. This has led to the increase of the Cronbach’s alpha of each scale by 0.06 to 0.09 points. Additionally, one item from each of the EOE and EUE factors were excluded to improve internal consistency (Cronbach’s alpha increased by 0.06 to 0.09 points); these items were “my child eats less when s/he is tired” and “my child eats more when s/he has nothing else to do,” respectively. Cronbach’s alphas for all factors ranged from 0.61 to 0.80. Cronbach’s alpha values of at least 0.60 are considered “good,” while values of at least 0.70 are considered “favorable”(Briggs and Cheek, 1986; DeVellis, 2003), Descriptive and internal consistency data are shown in Table 1.

**Table 1:** Descriptive and internal consistency data for child eating behavior and maternal indulgent feeding behavior factors.^a^

Factor	Mean	Standard deviation	Number of items	Cronbach’s Alpha
Child eating behaviors				
Emotional over eating	1.60	0.69	3	0.82
Emotional under eating	3.12	0.96	3	0.64
Food responsiveness	1.89	0.74	4	0.70
Enjoyment of food	3.02	0.81	3	0.79
Desire to drink	3.14	0.91	3	0.70
Food fussiness	3.30	0.84	6	0.80
Satiety responsiveness	3.51	0.79	4	0.66
Slowness in eating	3.29	0.77	4	0.61
Maternal indulgent feeding behaviors				
Use of food as a reward	2.21	1.02	4	0.74
Concern about child’s diet	2.26	1.20	7	0.85

a Internal consistency reflected by Cronbach’s Alpha. Cronbach’s alpha values of 0.60 are good and those of values of 0.70 or higher are favorable.

Mothers completed questions regarding the use of food as a reward and concern about the child’s diet, which reflected maternal behaviors at the time of the study when the child was between three and 5 years of age. The two scales, “use of food as a reward” and concern about child’s diet,” were adopted from the Child Feeding Questionnaire-Arabic (CFQ-A; Mosli, 2020). The CFQ-A is a version of the CFQ (Birch et al., 2001) which was translated into Arabic, modified, and validated among mothers of Saudi preschoolers (Mosli, 2020). The “use of food as a reward” scale consists of four items (e.g., “I give my child food to reward him/her for good behavior”) with response options ranging from 1 = “never” to 5 = “very often.” The “concern about child’s diet” scale consists of seven items (e.g., “child is not eating enough”) with response options ranging from 1 = “not at all concerned” to 6 = “extremely concerned” (Descriptive and internal consistency data are shown in Table 1).

#### 2.2.2. Predictors: early feeding practices

Mothers were asked to answer the following questions: “Did your child ever drink FJ from a baby bottle?” and “Did your child ever drink DSMM from a baby bottle?” Response options for these questions were: never; rarely; sometimes; most of the time; always. These variables were later dichotomized into “ever offered FJ in a baby bottle when child was an infant (yes vs. no)” and “ever offered DSMM in a baby bottle when child was an infant (yes vs. no).”

#### 2.2.3. Demographic characteristics

Characteristics assessed include child characteristics, such as child’s sex (male vs. female), birthdate (used to calculate child’s age), and birthweight [in kilograms (kg)]. Questions also assessed maternal educational level (middle school, high school, college, and postgraduate studies), and the family’s total income per month (later categorized into ≤10,000 SR vs. > 10,000 SR) [10,000 SR is equivalent to 2,666 USD and is considered a threshold for poverty in SA (Garawi et al., 2015)].

### 2.3. Statistical analysis

Analyses were completed using IBM SPSS Statistics 21.0 (Armonk, NY, United States). Sample characteristics were examined using descriptive statistics; Counts and percentages were calculated for categorical variables and means and standard deviations (SD) were calculated for continuous variables. Bivariate associations of early feeding practices with child eating behaviors and maternal indulgent feeding behaviors were examined using Spearman correlations. Child eating behaviors and maternal indulgent feeding behavior variables that were significantly correlated with early feeding practices were subsequently selected as endogenous variables in regression models. Prior to running regression models, we examined the distribution of child eating behavior and maternal indulgent feeding behavior variables. Kolmogorov Smirnov test *p*-value for our 3 dependent variables: “food responsiveness,” “food fussiness,” and “use of food as a reward” was <0.01, indicating that the data were not normally-distributed. We subsequently log-transformed the 3 dependent variables and reran the Kolmogorov Smirnov test. Results showed that only “food fussiness” was normalized (value of *p* = 0.20) while the *p*-value for “food responsiveness” and “use of food as a reward” remained <0.01, indicating lack of normality. Therefore, these dependent variables were used as dichotomous variables in logistic regression analysis as previously employed (DiSantis et al., 2011; Velders et al., 2012). The cut-off for categorizing the dependent variables was set at the highest tertile of the factor scores. Odds ratios (ORs) and respective 95% confidence intervals (CI) were derived from the logistic regression analysis. Both unadjusted models and models adjusted for child sex, child birthweight, maternal education, and total monthly income were examined. Each of these covariates has been previously associated with both maternal feeding behaviors and child eating behaviors (Birch and Davison, 2001; Syrad et al., 2016; Albuquerque et al., 2017; Jansen et al., 2018).For all statistical tests, significance level was set at *α* < 0.05.

## 3. Results

### 3.1. Sample characteristic

About 59% of children in the study were male, and mean child age was 4.91 (SD = 0.78) years. Mean child birthweight was 2.87 kg (SD = 0.68), and 68.7% of mothers had a college education. About half (54.8%) of participants reported a total monthly income of at least 10,000 SR (Table 2).

**Table 2:** Sample characteristics.^a^

Variables	Total (*n* = 115)
Child Sex, *N* (%)	
Male	68 (59.1)
Female	47 (40.9)
Child Age, M (SD)	4.91 (0.78)
Child birthweight (kg), M (SD)	2.87 (0.68)
Maternal Education, *N* (%)	
Middle School	2 (1.70)
High School	21 (18.3)
College	79 (68.7)
Postgraduate studies	13 (11.3)
Total family monthly income, *N* (%)	
< 10,000 SR	52 (45.2)
≥10,000 ΣΡ	63 (54.8)

a Table showing distribution of child, maternal and demographic variables: Means (M) and standard deviations (SD) for continuous variables or counts (*n*) and percentages (%) for categorical variables.

### 3.2. Correlation of early feeding practices with child eating behaviors and maternal indulgent feeding behaviors

Bivariate analysis showed a positive correlation between offering FJ in a baby bottle when child was an infant and child food responsiveness at preschool (*r* = 0.19, *p*-value = 0.04). Furthermore, there was a positive correlation between offering FJ in a baby bottle when child was an infant and maternal use of food as a reward at preschool (*r* = 0.19, *p*-value = 0.04). Offering DSMM in a baby bottle when child was an infant was associated with lower child food fussiness at preschool (*r* = −0.19, *p*-value = 0.04). There was no correlation between offering DSMM in a baby bottle when child was an infant and maternal indulgent feeding behaviors at preschool (Table 3).

**Table 3:** Correlation of early feeding practices with child eating behaviors and maternal indulgent feeding behaviors.^a^

	Child eating behaviors								Maternal indulgent feeding behaviors	
	Emotional over eating	Emotional under eating	Food responsiveness	Enjoyment of food	Desire to drink	Food fussiness	Satiety responsiveness	Slowness in eating	Use of food as a reward	Concern about child’s diet
Offering fruit juice in a baby bottle when child was an infant	0.10	0.13	0.19*	0.00	0.06	−0.04	0.11	−0.01	0.19*	0.04
Offering date-syrup milk mixture in a baby bottle when child was an infant	−0.09	0.03	0.02	−0.03	−0.16	−0.19*	−0.05	−0.02	−0.02	0.02

a Analysis performed using Spearman correlation.

^*^p-value < 0.05.

### 3.3. Regression analysis of the associations between early feeding practices and child eating behaviors

As shown in Table 4, children who were ever offered FJ in a baby bottle as infants were more likely to have high food responsiveness at preschool, compared to children who were not (OR = 2.76, 95% CI = 1.12, 6.82). This association did not meaningfully change after adjusting for child sex, child birthweight, maternal education, and total monthly income (OR = 2.93, 95% CI = 1.16, 7.42). The association between offering DSMM in a baby bottle when child was an infant and child food fussiness at preschool was not significant.

**Table 4:** Adjusted and unadjusted associations between early feeding practices and child eating behaviors.^a^

	Unadjusted odds ratio (95% CI)	Adjusted odds ratio (95% CI)^b^
*Food responsiveness*		
Ever offered fruit juice in a baby bottle when child was an infant		
Yes	2.76 (1.12, 6.82)^*^	2.93 (1.16, 7.42)^*^
No	1	1
*Food fussiness*		
Ever offered date-syrup milk mixture in a baby bottle when child was an infant		
Yes	0.61 (0.28, 1.33)	0.61 (0.27, 1.37)
No	1	1

a Associations examined using logistic regression.

b Regression models adjusted for child sex, child birthweight, maternal education, and total family monthly income.

^*^p-value <0.05.

CI, confidence interval.

### 3.4. Regression analysis of the associations between early feeding practices and maternal indulgent feeding behaviors

Mothers who reported ever offering FJ in a baby bottle when their children were more likely to adopt greater use of food as a reward when their children were at preschool (OR = 2.87, 95% CI = 1.11, 7.37). This association did not meaningfully change after adjusting for child sex, child birthweight, maternal education, and total monthly income (OR = 3.25, 95% CI = 1.22, 8.68; Table 5).

**Table 5:** Adjusted and unadjusted associations between early feeding practices and maternal indulgent feeding behaviors.^a^

	Use of food as a reward	
	Unadjusted odds ratio (95% CI)	Adjusted odds ratio (95% CI)^b^
Ever offered fruit juice in a baby bottle when child was an infant		
Yes	2.87 (1.11, 7.37)^*^	3.25 (1.22, 8.68)^*^
No	1	1

a Associations examined using logistic regression.

b Regression models adjusted for child sex, child birthweight, maternal education, and total family monthly income.

^*^p-value <0.05.

CI, confidence interval.

## 4. Discussion

Our present study found that children who were offered FJ in a baby bottle as infants were more likely to have higher food responsiveness at preschool age. This association was not attenuated after accounting for potential confounders. Our findings are consistent with previous work that showed that unhealthy feeding patterns during infancy may increase food responsiveness later in childhood (Daniels et al., 2014). Previous work has also suggested that maladaptive feeding practices during infancy may negatively influence appetite control and learn food pleasure among children (Nicklaus, 2017) Food responsiveness has been positively linked to obesogenic eating patterns and higher weight status among children (Carnell and Wardle, 2008; Syrad et al., 2016). Furthermore, we found through bivariate analysis, a negative correlation between offering DSMM in a baby bottle and child food fussiness at preschool. However, the regression analysis did not detect a significant association. Lower food fussiness has been associated with higher odds of overweight or obesity at preschool (Mosli et al., 2015), which may be explained by children accepting and consuming a greater variety and quantity of food.

Mothers who reported offering FJ in a baby bottle were more likely to adopt greater use of food as a reward when their children were between three and five years of age. Use of food as a reward has been found to lead to diminished ability to self-regulate and subsequent emotional overeating and higher weight status among children (Powell et al., 2017; Souto-Gallardo et al., 2020). Although we could not identify any previous studies that examined the association between offering sugary drinks during infancy and later maternal feeding behaviors, findings from earlier studies suggest that healthier feeding practices during infancy, such as breastfeeding for a longer duration, is associated with more responsive maternal feeding behaviors and greater sensitivity to infant satiety cues (Ventura, 2017). On the contrary, greater bottle-feeding intensity has been linked to maladaptive maternal feeding behaviors (e.g., use of pressure to eat) at age six (Li et al., 2014).

At present, offering FJ in a baby bottle may be a common practice among mothers in SA (Mosli, 2022). Since our cross-sectional study has detected a positive association between offering FJ in a baby bottle and food responsiveness at preschool, prospective research is needed to establish causality. Future studies may also focus on the evaluation of interventions targeting young children who were offered FJ in a baby bottle as infants and assessing the effect of these interventions on food responsiveness. Community awareness campaigns and education programs for mothers during prenatal and early postpartum stages regarding appropriate child feeding strategies and nutritional requirements are warranted. Furthermore, since we found that mothers who offered FJ in a baby bottle when their children were infants were more likely to use food as a reward later in childhood, mothers who reported using maladaptive early feeding practices may be specifically targeted for education and intervention programs to promote more appropriate feeding strategies as children grow. Previous research found that parents who ate less intuitively and did not rely on their own hunger and satiety cues in eating were more likely to practice indulgent feeding styles with their infants (Khalsa et al., 2019). Further work may focus on further understanding determinants of parental feeding practices during infancy.

Strengths of this study include that child eating and maternal feeding behaviors were assessed using questionnaires that have previous evidence of validity and reliability in a similar sample (Alhamad, 2013; Mosli, 2020). Furthermore, recruitment of participants was conducted at various locations in Jeddah, SA. Limitations of this study include that the sample size was relatively small which might have increased the possibility of type 2 error. We have included child eating behavior and maternal indulgent feeding behavior variables as dichotomous dependent variables rather than continuous variables in regression models, and we were therefore unable to evaluate the linear relationship of early feeding practices with eating behaviors among children and maternal indulgent feeding behaviors. Our cross-sectional design does not enable us to establish cause and effect. Additionally, since data regarding early feeding practices were gathered retrospectively, we cannot exclude the possibility of recall bias. Social desirability bias may have also been introduced through maternal report.

## 5. Conclusion

Findings from our study suggest that early feeding practices are associated with child eating and maternal feeding behaviors later in childhood. Future longitudinal studies with larger sample sizes are needed to further establish these associations. Different strategies for collecting eating and feeding behavior data, such as direct observation and mix-method approaches, may be useful. Additionally, future work may include nutritional status and growth data and an evaluation of how early feeding and eating behaviors relate to these outcomes. Community awareness and education programs are needed for mothers during prenatal and early postpartum stages in order to prevent maladaptive feeding practices and promote appropriate feeding strategies throughout childhood.

## Data availability statement

The raw data supporting the conclusions of this article will be made available by the authors, without undue reservation.

## Ethics statement

The studies involving human participants were reviewed and approved by from Unit of Biomedical Ethics at King Abdulaziz University. The patients/participants provided their written informed consent to participate in this study.

## Author contributions

RM: conceptualization, software, visualization, writing—original draft preparation, project administration, and funding acquisition. RM and HK: methodology, validation, formal analysis, investigation, resources, data curation, writing—review and editing, and supervision. All authors contributed to the article and approved the submitted version.

## Funding

This project was funded by the Deanship of Scientific Research (DSR) at King Abdulaziz University, Jeddah, under grant no J-734-142-38.

## Conflict of interest

The authors declare that the research was conducted in the absence of any commercial or financial relationships that could be construed as a potential conflict of interest.

## Publisher’s note

All claims expressed in this article are solely those of the authors and do not necessarily represent those of their affiliated organizations, or those of the publisher, the editors and the reviewers. Any product that may be evaluated in this article, or claim that may be made by its manufacturer, is not guaranteed or endorsed by the publisher.
